# Integrative Analysis of DNA Methylation and Gene Expression to Determine Specific Diagnostic Biomarkers and Prognostic Biomarkers of Breast Cancer

**DOI:** 10.3389/fcell.2020.529386

**Published:** 2020-12-07

**Authors:** Ming Zhang, Yilin Wang, Yan Wang, Longyang Jiang, Xueping Li, Hua Gao, Minjie Wei, Lin Zhao

**Affiliations:** ^1^Department of Pharmacology, School of Pharmacy, China Medical University, Shenyang, China; ^2^Liaoning Key Laboratory of Molecular Targeted Anti-tumor Drug Development and Evaluation, China Medical University, Shenyang, China; ^3^Liaoning Cancer Immune Peptide Drug Engineering Technology Research Center, China Medical University, Shenyang, China; ^4^Key Laboratory of Precision Diagnosis and Treatment of Gastrointestinal Tumors, Ministry of Education, China Medical University, Shenyang, China

**Keywords:** breast cancer, DNA methylation, DMSs, specific diagnostic biomarkers, prognostic markers, risk stratification

## Abstract

**Background:** DNA methylation is a common event in the early development of various tumors, including breast cancer (BRCA), which has been studies as potential tumor biomarkers. Although previous studies have reported a cluster of aberrant promoter methylation changes in BRCA, none of these research groups have proved the specificity of these DNA methylation changes. Here we aimed to identify specific DNA methylation signatures in BRCA which can be used as diagnostic and prognostic markers.

**Methods:** Differentially methylated sites were identified using the Cancer Genome Atlas (TCGA) BRCA data set. We screened for BRCA-differential methylation by comparing methylation profiles of BRCA patients, healthy breast biopsies and blood samples. These differential methylated sites were compared to nine main cancer samples to identify BRCA specific methylated sites. A BayesNet model was built to distinguish BRCA patients from healthy donors. The model was validated using three Gene Expression Omnibus (GEO) independent data sets. In addition, we also carried out the Cox regression analysis to identify DNA methylation markers which are significantly related to the overall survival (OS) rate of BRCA patients and verified them in the validation cohort.

**Results:** We identified seven differentially methylated sites (DMSs) that were highly correlated with cell cycle as potential specific diagnostic biomarkers for BRCA patients. The combination of 7 DMSs achieved ~94% sensitivity in predicting BRCA, ~95% specificity comparing healthy vs. cancer samples, and ~88% specificity in excluding other cancers. The 7 DMSs were highly correlated with cell cycle. We also identified 6 methylation sites that are highly correlated with the OS of BRCA patients and can be used to accurately predict the survival of BRCA patients (training cohort: likelihood ratio = 70.25, *p* = 3.633 × 10^−13^, area under the curve (AUC) = 0.784; validation cohort: AUC = 0.734). Stratification analysis by age, clinical stage, Tumor types, and chemotherapy retained statistical significance.

**Conclusion:** In summary, our study demonstrated the role of methylation profiles in the diagnosis and prognosis of BRCA. This signature is superior to currently published methylation markers for diagnosis and prognosis for BRCA patients. It can be used as promising biomarkers for early diagnosis and prognosis of BRCA.

## Introduction

Globally, breast cancer (BRCA) is currently the most common malignant cancer in women (Bray et al., 2018). Early detection of BRCA can significantly increase the chance of effective treatment and has a very important role in improving survival. If patients are diagnosed early, the 5-year survival rate is >90%, while the 5-year survival rate for patients with advanced BRCA is reduced to ~25% (Cardoso et al., 2018). From this, early detection of BRCA can increase the chance of effective treatment and has a very important role in improving survival. Cancer antigen 125 (CA125) is an ovarian-associated antigen found in tumors such as ovarian epithelial cancer, endometrial cancer, and breast cancer (Wang et al., 2017; Russell et al., 2019; Zang et al., 2019), which has been used as a diagnostic marker of breast cancer. The expression levels of bone sialoprotein (BSP) and osteopontin (OPN) serve as markers for lung cancer, breast cancer and prostate cancer (Fedarko et al., 2001). However, CA125 has a specificity of 97.0% in the diagnosis of breast cancer, but its sensitivity is relatively low at 25.6%. BSP (sensitivity 88.9%, specificity 96.1%) and OPN (sensitivity 95.0%, specificity 84.5%) can achieve a high accuracy rate for the diagnosis of breast cancer. But their diagnostic threshold is very close to other tumors. Therefore, it is very important to find specific diagnostic markers for breast cancer.

Studies have shown that DNA methylation abnormality, an epigenetic modification, is closely related to the occurrence and development of cancer (Hahn and Weinberg, 2002; Gu et al., 2006; Shen et al., 2017; Guo et al., 2019). The changes of DNA methylation have been observed in various types of cancers (Maruya et al., 2004; Aine et al., 2015; Nguyen et al., 2017; Bian et al., 2018; Jurmeister et al., 2019; Majumder et al., 2019; Norgaard et al., 2019). There are two patterns of cancer gene methylation which are related to cancer occurrence: genome-wide hypomethylation and promoter domain CpG island hypermethylation (Cheng et al., 2018). DNA methylation affects genes involved in different cellular pathways, Including cell proliferation, invasion and apoptosis (Gopisetty et al., 2006; Shao et al., 2018). In addition, DNA molecules in tumor cells are released into the blood as a result of apoptosis or necrosis as cell-free tumor DNA (ctDNA), where the DNA methylation of ctDNA in cancer patients have been found to be different from that in healthy individuals (Visvanathan et al., 2017). Therefore, the methylation detection of ctDNA in the blood can be used for cancer detection (Xu et al., 2017). More and more biomarkers based on methylation have been developed to help early diagnosis of cancer (Shen et al., 2017; Cheng et al., 2018; Toth et al., 2019). Wu et al. reported that DNA methylation with 4 CpGs can distinguish the healthy people and BRCA patients, with a sensitivity of over 97% and a specificity of nearly 91% (Wu et al., 2019). Core et al. found that methylation can also distinguish between BRCA patients and healthy people, with a sensitivity of more than 83% and a specificity of more than 90% (Croes et al., 2018). Both markers are good biomarkers for diagnosing BRCA. However, cancers are heterogeneous diseases, none of them considered whether other types of cancer had similar methylation changes. In this study, we identified 7 BRCA-specific methylation biomarkers by comparing BRCA to normal breast and other cancer types. And we also identified 6 CpG sites that could predict the survival of BRCA patients.

## Materials and Methods

### Analysis of DNA Methylation and Gene Expression Differences

DNA methylation, gene expression, and clinical BRCA data are from the cancer genome atlas (TCGA) (International Cancer Genome Consortium et al., 2010). The data are downloaded from UCSC Xena (http://xena.ucsc.edu). DNA methylation profile was measured experimentally using the Illumina Infinium HumanMethylation 450 platform which contains 485,577 CpG sites. The methylation level is expressed as β value. Poor performing probes, cross reactive probes, Y chromosomes probes and SNP probes have been excluded in our data processing. Because the vast majority of breast cancer patients are female, the X chromosomes probes have not been excluded. R function “normalizeBetweenArrays” was be used to normalize the data between arrays function. Methylation data of another 9 [Glioblastoma (two normal, 153 cancer), Bladder Cancer (21 normal, 413 cancer), Liver Cancer (50 normal, 379 cancer), Head and Neck Cancer (50 normal, 530 cancer), Cervical Cancer (three normal, 309 cancer), Lung Adenocarcinoma (32 normal, 460 cancer), Lung Squamous Cell Carcinoma (43 normal, 372 cancer), Colon Cancer (38 normal, 309 cancer) and Rectal Cancer (seven normal, 99 cancer) cancer tissues and adjacent tissues were collected from the TCGA, and the 184 blood samples of healthy people were collected from the database GSE69270 (the profile of these cases in the Supplementary Table 1). Excluded sites were related to gender (male and female, |Δβ| > 0.2, FDR < 0.05) (Supplementary Table 2). The β values of methylation sites with missing values over 10% were deleted. The remaining missing values were estimated by the k-Nearest Neighbor (KNN) estimation method. The “limma” package(Ritchie et al., 2015) was used to calculate the methylation difference. The sites with an FDR < 0.05 and an absolute of the β value difference >0.2 were considered to be differentially methylated. The gene expression profile was measured experimentally using the Illumina HiSeq 2000 RNA Sequencing platform. For the correlation analysis of DNA methylation and gene expression, we used the R package “ChAMP” to map sites assigned to a gene. Pearson correlation test was used to obtain the correlation between them. The correlation coefficient >0.3 and the *p*-value < 0.05 were considered to be significant. The correlation coefficient of DMSs was obtained by Pearson correlation test, and R package “corplot” was used to plot the correlation between DMSs.

### Evaluation of Candidate Diagnostic Biomarkers

The TCGA breast cancer DNA methylation data were randomly sorted. Sixty seven percent of them (515 tumor tissues, 77 normal tissues) were used as training cohort and 33% (275 tumor tissues, 21 normal tissues) were used as validation cohort. The Wilcox test was used to find differential methylated sites (DMSs) in the training cohort (|Δβ|> 0.2, FDR < 0.05). Next, Pearson correlation test was performed on these DMSs and their corresponding genes to find sites that can drive gene expression. Then, Wilcox test was used to screen the DMSs in breast cancer samples and normal blood samples (|Δβ|> 0.2, FDR < 0.05) to eliminate the interference factors of blood. Next, these DMSs that could drive gene expression were subjected to Wilcox test (|Δβ| > 0.2, FDR < 0.05) in breast cancer and other nine tumors and para-cancerous tissues to discover breast cancer specific diagnostic biomarkers. Finally, we used the WrapperSubsetEval evaluator, which used cross-validation to evaluate the accuracy of each subset's learning scheme to assess the predictive power of each DMS and select the most representative of the DMSs as diagnostic biomarkers. The BaysNet model was built using the Weka software (version 3.8 at https://waikato.github.io/weka-wiki/downloading_weka/). Weka is a machine learning software which tries and tests open source. Our goal was to build a classifier from sample information with known histological characteristics (whether it is BRCA tissue) and use the classifier to predict whether the sample to be tested is BRCA tissue. We constructed a classifier based on the β value of the BRCA-specific DMSs of the training cohort. The classifier compares the characteristics of the DMSs in BRCA tissues and BRCA para-cancerous tissues. Then we learned various thresholds or rules and stored them in the constructed classifier. For learning Bayesian network, we leveraged the K2 algorithm (Lerner and Malka, 2011). Three other independent data sets GSE66695, GSE60185 (Fleischer et al., 2014) and GSE78754 (Mathe et al., 2016) are used as external test cohort. We also organized the profile of the three external test cohort (Supplementary Tables 3–5).

### Prognostic Marker Selection

Prognostic markers were selected from 776 BRCA patients with methylation data and clinical information. They are shuffled and randomly reordered. Sixty seven percent of them were the training cohort (517 cases) and 33% of them were the validation cohort (259 cases). The univariate Cox proportional hazard analysis was performed in the training cohort to find the methylation sites significantly related to the survival of patients. Then, in univariate analysis, the sites that were significantly related to OS were included in the multivariate Cox regression analysis, and a model containing all possible combinations of 2 to 6 factors was constructed to select the best combination of biomarkers. The R-package “mass” was used for analysis.

### Gene Ontology Enrichment Analysis and Pathway Enrichment Analyzes for Diagnostic Biomarkers

PPI (protein protein interaction) network, KEGG (Kyoto Encyclopedia of Genes and Genomes) pathway analysis, and GO (Gene Ontology) pathway were analyzed by the STRING database. Line color indicated the type of interaction evidence, minimum required interaction score was 0.70. The R-package “ggplot” and “GOplot” was used for plotting.

### GSEA Enrichment Analysis for Prognostic Biomarkers

In order to explore the biological pathways of CpG markers, Gene Set Enrichment Analysis (GSEA) was used (Subramanian et al., 2005). The annotated c2.cp.kegg.v6.2.symbols.gmt gene set is regarded as the reference gene set. The critical criterion was *p*-value < 0.05 and *q*-value < 0.25

### The Gene Expression Omnibus Dataset

Four DNA methylation datasets were collected from the Gene Expression Omnibus (GEO) database: GSE66695 (80 breast cancer, 40 normal), GSE78754 (Mathe et al., 2016) (80 breast cancer), GSE60185 (Fleischer et al., 2014) (239 breast cancer, 46 normal), and GSE69270 (Kananen et al., 2016) (normal blood).

### Immunohistochemistry

The assay was carried out according to the method mentioned in the previous study (Song et al., 2020). We collected breast cancer tissues of patients from the First Affiliated Hospital of China Medical University for Immunohistochemistry (IHC) assay. The IHC antibodys were ordered from BOSTER Biological Technology co.ltd (USA).

### Statistical Analysis

Differential methylation calculated from mean (β value- cancer)–mean (β-value- normal). Wilcox test was used to determine the different methylation sites between tumor and normal tissues. Use the false discovery rate (FDR) method to adjust the *p*-value. The sites with an absolute difference of β-value > 0.2 and FDR < 0.05 are considered to be differentially methylated. The hazard ratio (HR) and the corresponding 95% confidence interval (CI) were evaluated by the Cox proportional risk model. The ROC analysis was performed by the proc package to determine the area under the curve (AUC). All data analyses were performed with R (R version 3.5.4).

## Results

### Analysis of Differential Methylation Profiles and Identification of Candidate CpG Sites for BRCA Specific Diagnosis

**Figure 1:**
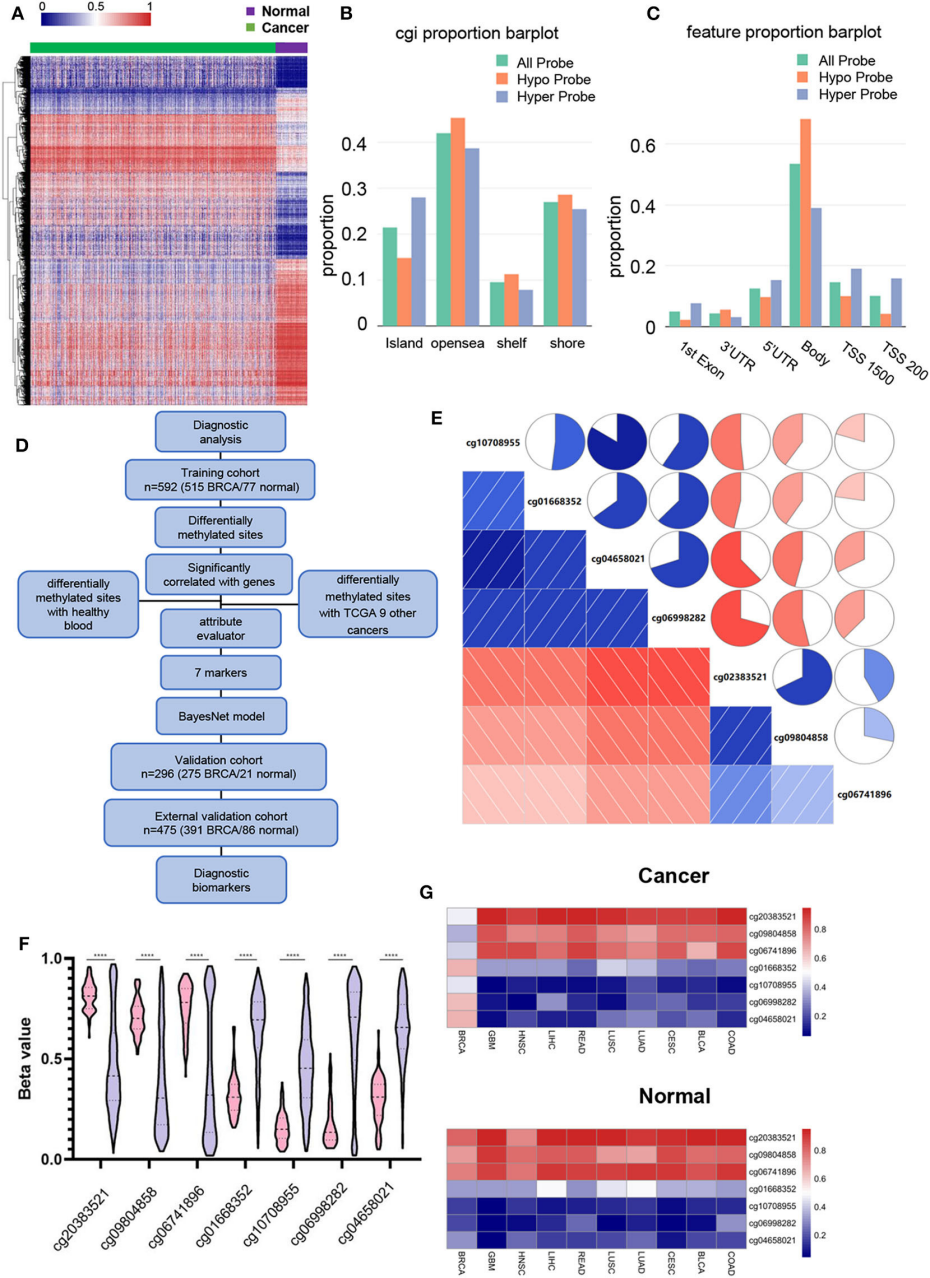
Identifying BRCA-specific differentially methylated sites. **(A)** Heatmap of the differentially methylated sites, contains 2,362 hypermethylated and 2,322 Hypomethylated DMSs. Green are cancer tissues and purple are normal tissues. **(B)** Differential methylation sites distribution in CpG island, opensea, shelf, and shore. The DNA methylation sites between genes have been omitted. **(C)** Differentially methylated the distribution of DMS based on the distance to the TSS. **(D)** Flowchart for finding BRCA candidate diagnostic biomarkers. **(E)** Correlation between 7 DMSs. The square and circle symbols represent the one-to-one correlation coefficient. Blue indicated the positive correlation, and red indicated the negative correlation. Each correlation coefficient was shown by the shadow intensity and increased uniformly as the correlation value starts from 0 to 1. **(F)** Methylation level of 7 DMSs in BRCA and normal tissues. Pink represents normal tissue, purple represents BRCA tissue. **(G)** The sites mean β value show the methylation levels of 7 BRCA markers in BRCA and nine other cancers.

DNA methylation of 790 BRCA tumor samples and 98 adjacent normal tissue samples obtained from TCGA were used for differential methylation analysis. Sixty seven percent of the samples were used as training cohort, and 33% were used as validation cohort (Table 1). The Wilcox test was used to find differential methylated sites (DMSs) in the training cohort (|Δβ|> 0.2, FDR < 0.05). Pearson correlation test was performed on these DMSs to find the DMSs that can drive gene expression (| r |> 0.3, *p* < 0.05) (Supplementary Table 6). There were 2,362 hypermethylated and 2,322 Hypomethylated DMSs in BRCA (Figure 1A), which correspond to 1,157 hypermethylation and 989 hypomethylated genes. We then analyzed the distribution of DMSs in different genomic regions. The hypermethylation and hypomethylation sites were mainly located in the opensea, and the second hypermethylation sites were mainly located in the island, while the hypomethylation sites island, shore and shelf had obvious distribution (Figure 1B). From the point of view of gene distribution, DMSs were mainly distributed on the body, but the vicinity of the promoter region was mainly concentrated by hypermethylation sites. The distribution of hypomethylation sites was relatively broad (Figure 1C). This was consistent with the general characteristics of solid tumor DNA methylated.

**Table 1:** Clinicopathological characteristics of BRCA sample.

		**Sample**					
**Characteristics**	**Groups**	**Total (*****n*** **=** **888)**		**Training cohort**		**Validation cohort**	
				**(*****n*** **=** **592)**		**(*****n*** **=** **296)**	
		**No**	**%**	**No**	**%**	**No**	**%**
T-staging	T1-T2	653	73.54	430	72.64	223	75.34
	T3-T4	133	14.98	83	14.02	50	16.89
	Unknown	4	0.45	2	0.34	2	0.68
N-staging	N0-N1	624	70.27	403	68.07	221	74.66
	N2-N3	153	17.23	106	17.91	47	15.88
	Unknown	13	1.46	6	1.01	7	2.36
M-staging	M0	615	69.26	406	68.58	209	70.61
	M1	13	1.46	7	1.18	6	2.03
	Unknown	162	18.24	102	17.23	60	20.27
Stage	Stage I-II	568	63.96	369	62.33	199	67.23
	Stage III-IV	210	23.65	140	23.65	70	23.65
	Unknown	12	1.35	6	1.01	6	2.03
ER	Positive	575	64.75	373	63.01	202	68.24
	Negative	170	19.14	111	18.75	59	19.93
	Indeterminate or Not Evaluated	45	5.07	31	5.24	14	4.73
PR	Positive	502	56.53	332	56.08	170	57.43
	Negative	240	27.03	150	25.34	90	30.41
	Indeterminate or Not evaluated	48	5.41	33	5.57	15	5.07
HER2	Positive	93	10.47	61	10.30	32	10.81
	Negative	398	44.82	265	44.76	133	44.93
	Indeterminate or Not evaluated	299	33.67	189	31.93	110	37.16
Normal tissue	98	11.04	77	13.01	21	7.09	

In order to find the specific diagnostic biomarkers of BRCA, we designed a workflow (Figure 1D). Filtration was performed using methylation data from healthy human blood (GSE69270). Two thousand six hundred and forty three DMSs that were differentially methylated between BRCA (|Δβ| > 0.2, FDR < 0.05) and healthy individuals' blood were left. Secondly, we screened the differentially expressed DMSs of the above breast cancers with other nine common cancers and their corresponding adjacent tissues (|Δβ| > 0.2, FDR < 0.05). There were still 17 DMSs with methylation differences in BRCA and other cancers and adjacent tissues. Finally, we used the WrapperSubsetEval evaluator, which used cross-validation to evaluate the accuracy of each subset's learning scheme to assess the predictive power of each DMS and select the most representative of the 7 DMSs (cg20383521, cg09804858, cg06741896, cg01668352, cg10708955, cg06998282, cg04658021) (Table 2). The 7 DMSs had a significant correlation with the corresponding gene expression (Supplementary Figure 1). These 7 DMSs were significantly correlated with each other (*p* < 0.05), Among them, cg10708955 and cg04658021 had the strongest correlation (*r* = 0.803), indicating that the 7 DMSs might jointly mediate the occurrence of BRCA (Figure 1E). They were differentially methylated between BRCA and normal tissues (Figure 1F). Unsupervised cluster analysis revealed that BRCA samples were well-differentiated from normal tissues, indicating the robustness of our results (Supplementary Figure 2). Similarly, 7 DMSs were also different methylate between BRCA and other cancer tissues (Figure 1G).

**Table 2:** The 7 DMSs for BRCA diagnosis.

**CpG sites**	**Gene**	**Gene function**	**Feature type**
cg20383521	TUFT1	Tuftelin 1	opensea
cg09804858	TRERF1	Transcriptional regulating factor 1	opensea
cg06741896	CCND1	Cyclin D1	shore
cg01668352	SRGAP1	SLIT-ROBO Rho GTPase activating protein 1	opensea
cg10708955	PER1	Period circadian regulator 1	shore
cg06998282	ENPP2	Ectonucleotide pyrophosphatase/phosphodiesterase 2	opensea
cg04658021	PER1	Period circadian regulator 1	shore

### Evaluation of Diagnostic Accuracy in Independent Datasets

**Figure 2:**
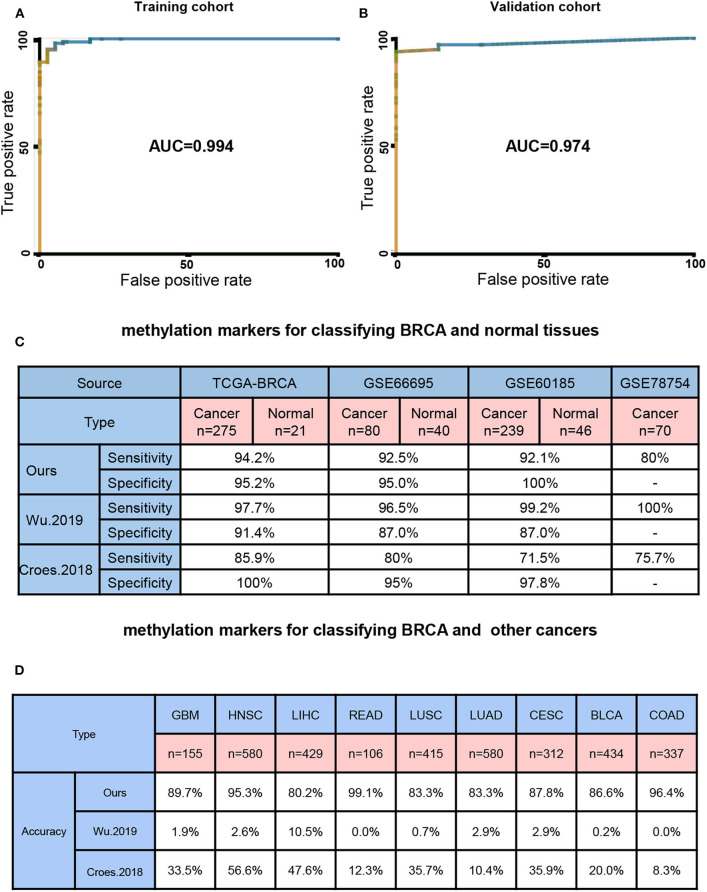
Seven BRCA-specific differential methylation sites as a diagnostic biomarker. **(A)** model training cohort ROC curve area measurement. **(B)** Model validation cohort ROC curve area measurement. **(C)** Four different source sets test the correct rate of the 7 DMSs model and other models. **(D)** Nine other cancers set test the 7 DMSs model and other models correct rate.

Next, we built a BayesNet model based on 7DMSs through the TCGA training cohort data. We tested the model accuracy in the TCGA validation cohort. Three independent methylation data sets (GSE66695, GSE60185, and GSE78754) of BRCA were used as external test sets. Our model had a training cohort of AUC = 0.994 and a validation cohort of AUC = 0.974 (Figures 2A,B). We then compared our results to previously published methylation markers, Wu et al. reported that DNA methylation with four CpGs could distinguish BRCA patients from healthy individuals (Wu et al., 2019). Core et al. found that methylation could also distinguish between BRCA patients and healthy people (Croes et al., 2018). The sensitivity and specificity of distinguishing between normal breast and breast cancer were high among different feature sets (Figure 2C). Next, we examined the ability of different methylation markers to distinguish between BRCA and other cancers. When our BRCA-specific markers were used in our study, few tumors and normal tissues of other cancers were predicted to be BRCA (0–19.8%, median 13.4%). However, 89.5–100% (median 98.1%) of other cancers and normal tissue were expected to be BRCA using markers from Wu et al. And markers from Croes et al. (2018) 43.4–91.7% (median 66.5%) of other cancers and normal tissue were expected to be BRCA (Figure 2D). Therefore, our study found highly specific biomarkers for BRCA diagnosis.

### Altered Functional Characteristics Related to the 7 DNA Methylation Signatures for BRCA Specific Diagnosis

**Figure 3:**
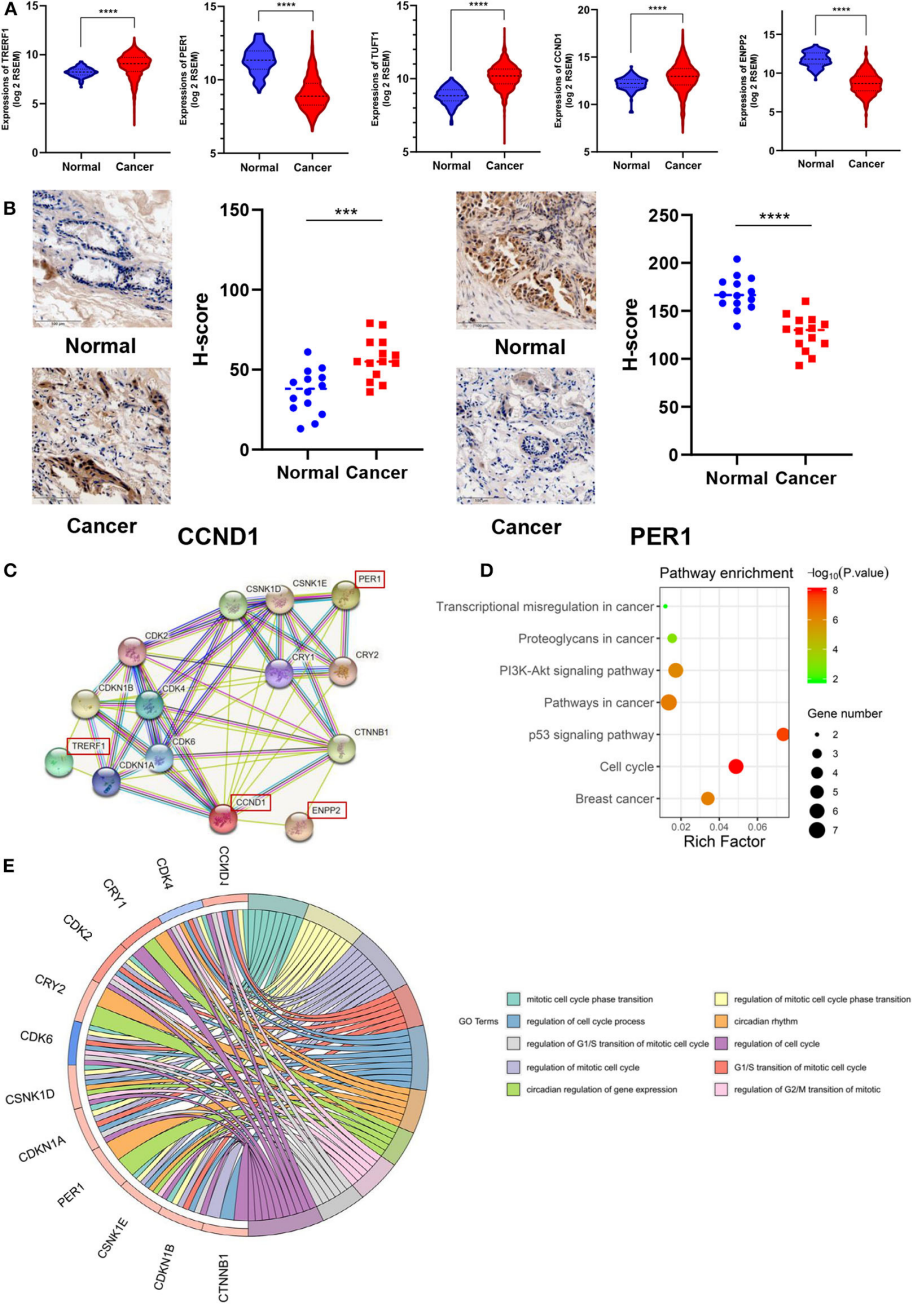
Association of 7 DNA methylation signatures with the cell cycle. **(A)** DMSs corresponding genes expression in TCGA. **(B)** CCND1 and PER1 protein expression in 14 paired BRCA and adjacent tissue by IHC assay. **(C)** PPI expression network for six genes corresponding to the seven methylation sites. **(D)** The KEGG pathway analysis of the mRNA of the PPI network map of the seven methylation sites corresponding gene. The vertical axis is the enriched pathway, and the horizontal axis is the number of genes enriched in this pathway compared to the number of genes on this pathway. **(E)** GO enrichment analysis. The circle indicates the correlation between the methylation-driven mRNAs and their gene ontology terms.

In order to further investigate the correlation between these 7 newly discovered DMSs and BRCA progression, we investigated whether their corresponding genes are differentially expressed in breast cancer in the TCGA gene expression data. The results shown that the expression of TRRERF1, PER1, TUFT1, CCND1, and ENPP2 genes was significantly different in breast cancer and adjacent tissues (*p* < 0.0001) (Figure 3A). Considering potential clinical significance and biologic implications, we performed immunohistochemistry (IHC) to evaluate the expression of CCND1 and PER1 in 14 paired BRCA and adjacent tissue. The results confirmed that CCND1 was highly expressed in breast cancer tissues and PER1 was low expressed in breast cancer tissues, which is consistent with the results of our data analysis (Figure 3B). To explore the biological behavior that our markers may be involved in, we constructed a PPI expression network using the STRING database for 6 DNA methylation-driven genes mapped by the 7 DMSs. There were only four genes: TRERF1, CCND1, PER1 and ENPP2 forming networks, TUFT1 and SRGAP1 did not form networks with other genes. The other genes in the network were all reported to be related to these four genes (Figure 3C). KEGG pathway analysis indicated these pathways were significantly correlated with these genes: Cell cycle, p53 signaling pathway, Pathways in cancer, Breast cancer, PI3K-Akt signaling pathway, Proteoglycans in cancer, Transcriptional misregulation in cancer (*p* < 0.05) with the most significant related pathway was “Cell cycle” (*p* = 9.79 × 10^−09^) (Figure 3D). At the same time, GO pathway analysis shown that the mitotic cell cycle phase transition was significantly correlated with these genes (*p* < 0.05) (Figure 3E). Among them, a number of biological processes were related to the cell cycle. This suggests that our 7 DNA methylation signature may be involved in the regulation of the cell cycle.

### Identification of the Prognostic DNA Methylation Signature in BRCA

**Figure 4:**
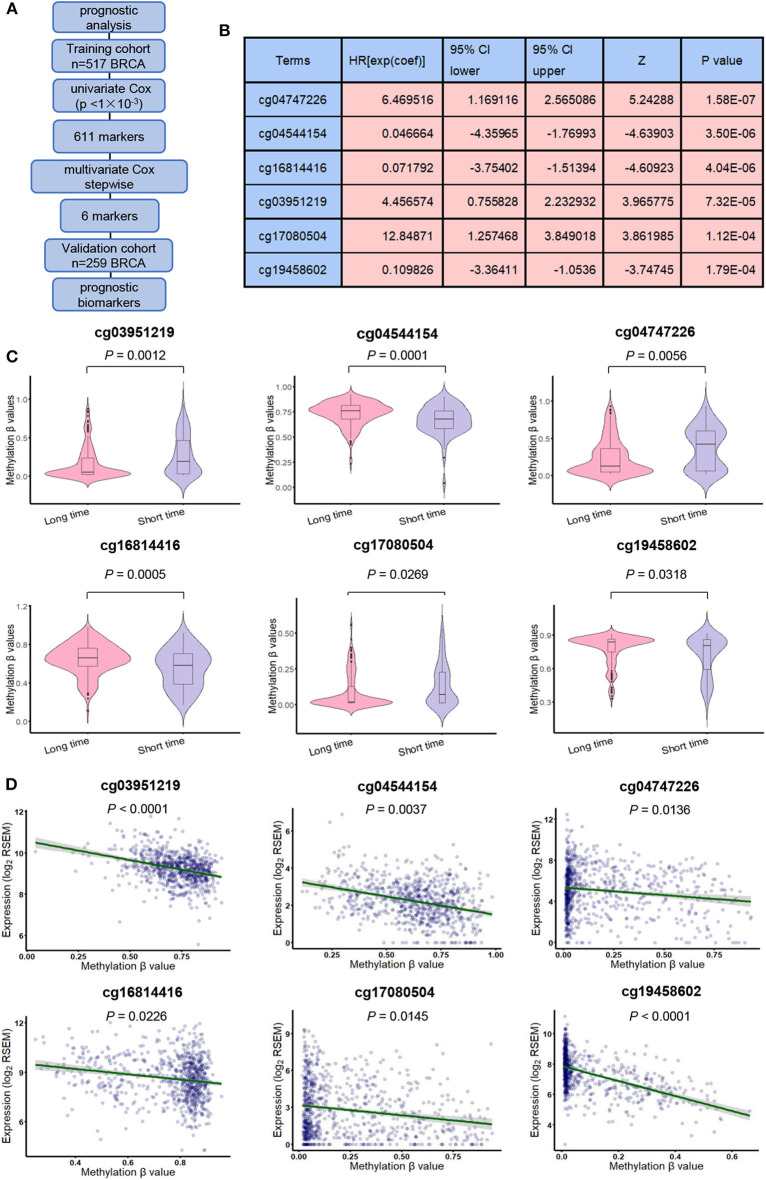
Derivation of prognostic DNA methylation markers. **(A)** The flowchart for finding BRCA candidate prognostic biomarkers. **(B)** General characteristics of univariate Cox regression analysis of six methylation biomarkers. **(C)** methylation β values in short survival (OS <5 years) patients and long survival (OS > 5 years) patients. **(D)** Pearson correlation test was used to evaluate the correlation between gene expression and methylation level.

We also designed a workflow to screen BRCA prognostic biomarkers (Figure 4A). The clinicopathological characteristics of BRCA patients in training cohort and validation cohort are summarized in Table 3. By performing a univariate Cox proportional hazards regression analysis in the training cohort, a total of 611 DNA methylation sites were significantly associated with OS in BRCA patients (*p* < 1 × 10^−3^), and they were used as candidate markers (Supplementary Table 7). Subsequently, these candidate markers were used to perform multivariate Cox stepwise regression analyses. Finally, the 6 methylation sites (cg04747226, cg04544154, cg16814416, cg03951219, cg17080504, cg19458602) was selected as the best prognosis model to predict OS (Figure 4B). The risk scoring formula was as follows:

**Table 3:** Clinicopathological characteristics of BRCA patients from TCGA database.

		**Patients**					
**Characteristics**	**Groups**	**Total (*****n*** **=** **776)**		**Training cohort (*****n*** **=** **517)**		**Validation cohort (*****n*** **=** **259)**	
		**No**	**%**	**No**	**%**	**No**	**%**
Age at diagnosis	≤58	401	51.68	248	47.97	135	52.12
	>58	375	48.32	269	52.03	124	47.88
T-staging	T1-T2	642	82.73	434	83.95	208	80.31
	T3-T4	131	16.88	80	15.47	51	19.69
	Unknown	3	0.39	3	0.58	0	0.00
N-staging	N0-N1	616	79.38	410	79.30	206	79.54
	N2-N3	150	19.33	101	19.54	49	18.92
	Unknown	10	1.29	6	1.16	4	1.54
M-staging	M0	611	78.74	394	76.21	212	81.85
	M1	13	1.68	11	2.13	2	0.77
	Unknown	152	19.59	112	21.66	45	17.37
Stage	stage I-II	560	72.16	373	72.15	187	72.20
	stage III-IV	205	26.42	133	25.73	72	27.80
	Unknown	11	1.42	11	2.13	0	0.00
ER	Positive	565	72.81	381	73.69	184	71.04
	Negative	168	21.65	112	21.66	56	21.62
	Indeterminate or Not Evaluated	43	5.54	24	4.64	19	7.34
PR	Positive	494	63.66	327	63.25	167	64.48
	Negative	236	30.41	164	31.72	72	27.80
	Indeterminate or Not Evaluated	46	5.93	26	5.03	20	7.72
HER2	Positive	92	11.86	55	10.64	37	14.29
	Negative	393	50.64	263	50.87	128	49.42
	Indeterminate or Not Evaluated	291	37.50	192	37.14	94	36.29
Metastasis	Yes	19	2.45	14	2.71	5	1.93
	No	757	97.55	503	97.29	254	98.07
Drug	Yes	583	75.13	387	74.85	196	75.68
	No	193	24.87	130	25.15	63	24.32
Race	white	565	72.81	370	71.57	195	75.29
	Black or African American	158	20.36	108	20.89	50	19.31
	Other	38	4.90	29	5.61	9	3.47
	Not Evaluated	15	1.93	10	1.93	5	1.93
Vital status	Alive	676	87.11	447	86.46	228	88.03
	Dead	101	13.02	70	13.54	31	11.97

RiskScore = 1.78920 × cg04747226–1.97075 × cg04544154–2.92310 × cg16814416 + 1.69264 × cg03951219 + 1.84526 × cg17080504–2.33118 × cg19458602.

The risk score indicates the chance of belonging to low-risk or high-risk group. Among the 6 methylation sites, cg04747226, cg03951219, and cg17080504 had positive correlation coefficients, indicating that their high DNA methylation level may be related to the short OS. cg04544154, cg16814416, and cg19458602 had negative correlation coefficients, indicating that their high DNA methylation level might be related to the longer OS. At the same time, for these 6 DNA methylation sites, DNA methylation levels were significantly different between patients exhibiting long-term (> 5 years) and short-term (<5 years) survival. cg04747226, cg03951219and cg17080504 shown long-term survival in patients who tended to have lower methylation levels, while cg04544154, cg16814416and cg19458602 shown long-term survival in patients who tend to have higher methylation levels (*p* < 0.05, Figure 4C). Then, we analyzed the correlation between these 6 methylation sites and their corresponding genes and found that they were significantly associated with the corresponding genes (*p* < 0.05, Figure 4D).

### The Prognostic Potential of 6 DNA Methylation Markers for BRCA Training and Validation Cohort

**Figure 5:**
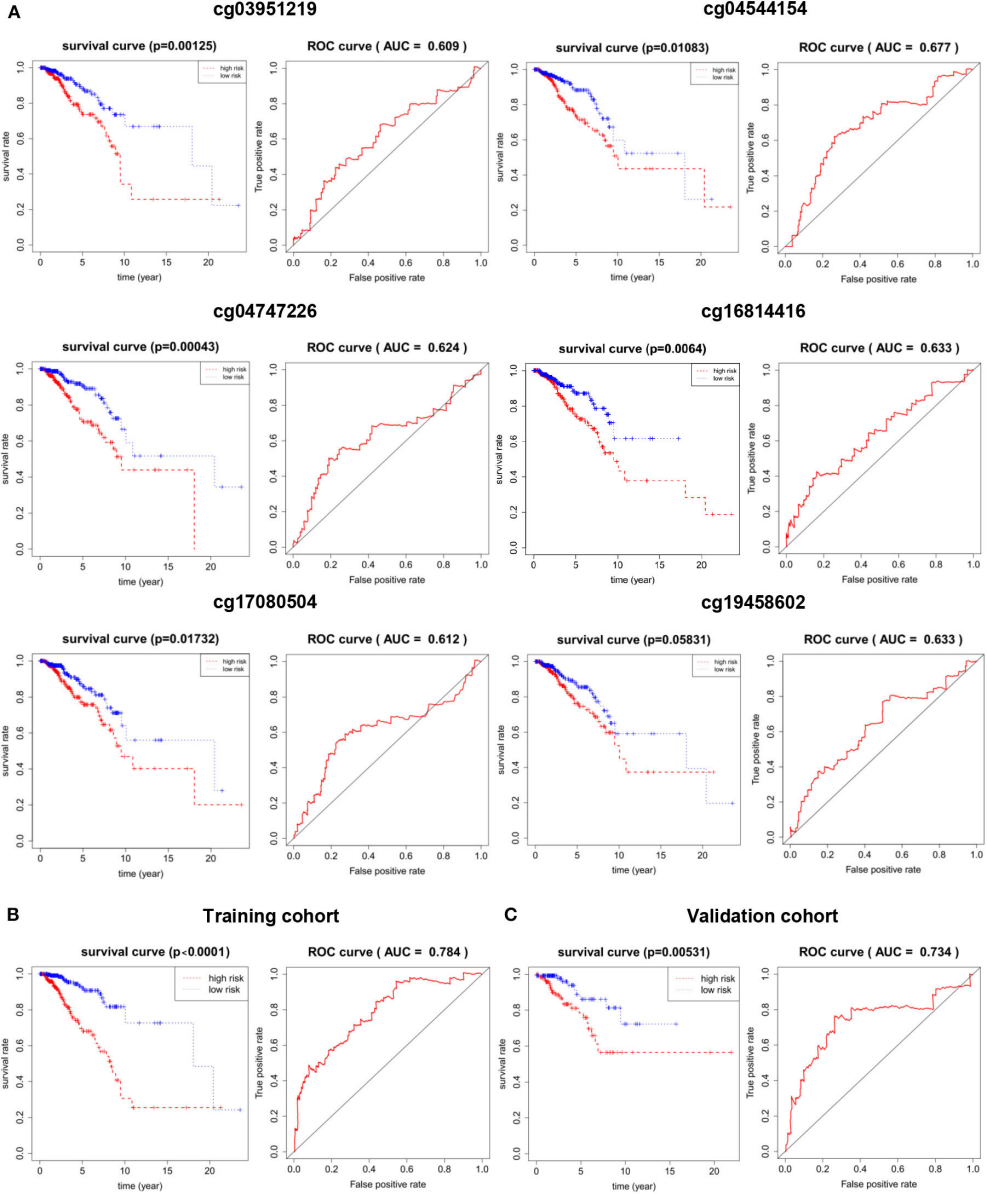
The prognostic potential of the 6 DNA methylation markers. **(A)** single site of methylation of breast cancer predicted and Kaplan-Meier survival analysis of the AUC of the ROC curve area. **(B)** Kaplan-Meier survival analysis and sensitivity and specificity for ROC analysis of predictive the power of 6 DNA methylation markers in predicting OS in patients in the training cohort. **(C)** Kaplan-Meier survival Analysis and ROC analysis in validation cohort.

In order to understand the accuracy of 6 DNA methylation markers in predicting the survival, the median of β-value of these sites were used as the cut-off point to distinguish between the high and low risk groups, the value of AUC was calculated by time-dependent ROC analysis, overall survival for outcome variable. In the validation cohort, Kaplan Meier survival analysis was performed on these six markers, and the AUC was calculated. The AUC of the 6 CpG sites in the validation set could reach more than 0.6, and the KM curve could effectively distinguish the high-risk and low-risk groups (Figure 5A). However, after combining them (the median of risk score as cutoff), the 6 DNA methylation markers had a good predictive ability for patient OS in the training and validation cohort. the AUC = 0.784 (Figure 5B) and 0.734 (Figure 5C), respectively. These results indicate that the six methylation markers have high sensitivity and specificity, the markers may have great potential in clinical application as prognostic biomarkers.

### In Terms of Clinical and Pathological Factors, the Independence of the 6 DNA Methylation Markers in OS Prediction

**Figure 6:**
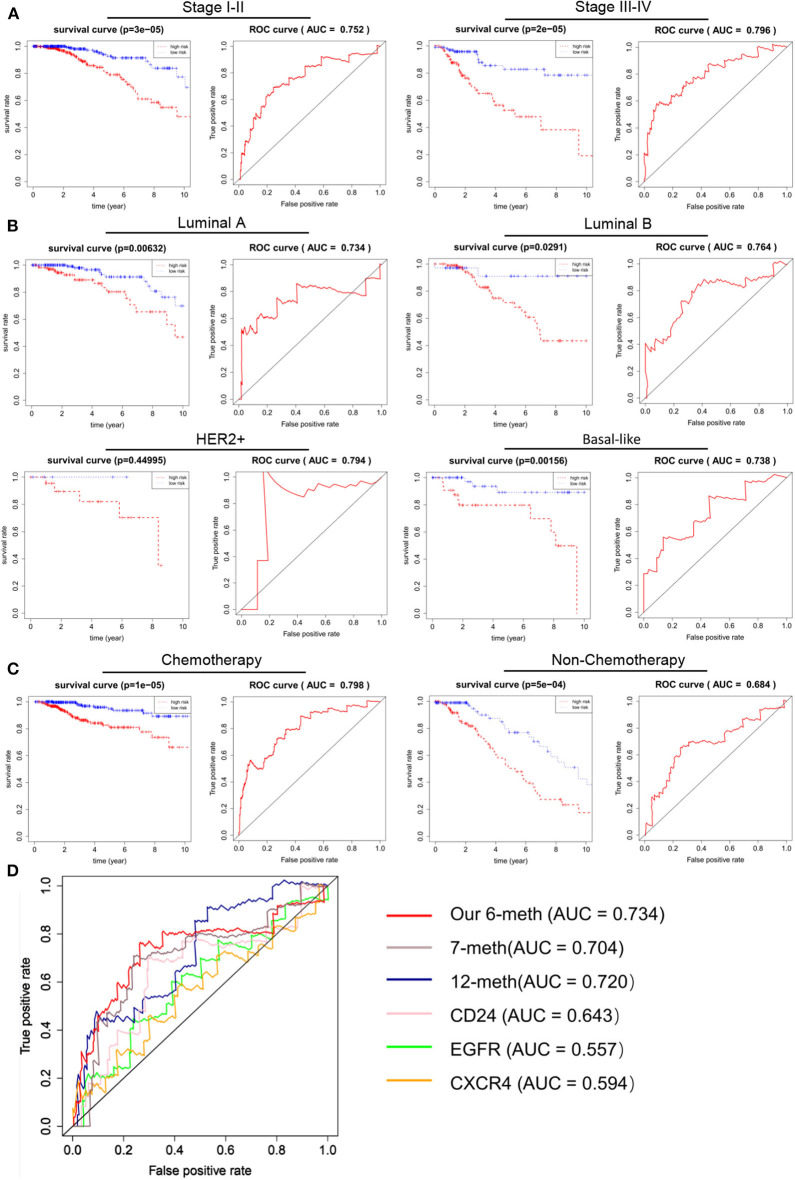
Independence of 6 DNA methylation markers in OS prediction, and comparison with reported markers. **A** Kaplan–Meier and ROC analysis were performed on patients with different stages of BRCA. Stage I-II (*N* = 560, 72.16%), Stage III-IV (*N* = 205, 26.42%). **(B)** Kaplan–Meier and ROC analyses were performed on BRCA patients with different phenotypes. According to their tumor phenotype, luminal A breast cancer (*N* = 277, 35.70%), luminal B breast cancer (*N* = 126, 16.24%), HER2+ breast cancer (*N* = 29, 3.74%) and basal-like breast cancer (*N* = 85, 10.95%). **(C)** Kaplan–Meier and ROC analysis were performed on BRCA patients with different treatment regimens. Grouped according to whether they were undergoing chemotherapy. After chemotherapy (*N* = 583, 75.13%), no chemotherapy (*N* = 193, 24.87%). **(D)** The ROC curve shows the sensitivity and specificity of our 6 DNA methylation markers and other known biomarkers in predicting patient OS based on the TCGA validation data set.

An excellent prognostic marker should be independent of the current clinical pathological prognostic indicators or be able to cooperate with them. Clinical and pathological features, such as patient age, clinical stage, and tumor classification, were also considered to be major predictors of prognosis in breast cancer patients. In order to evaluate the independence and applicability of 6 DNA methylation markers, patients were recombined according to different clinicopathological characteristics. Considering that the clinical staging, type, and clinical medication of BRCA can affect the prognosis, we regrouped patients based on different clinical characteristics. According to the progress of breast cancer, we divided the patients into early stage (stage I-II) and advanced stage (stage III-IV). Although the disease progression of these two groups of patients was markedly different, the OS between high-risk and low-risk populations are significantly different (*p* < 1 × 10^−3^). AUC of the early and advanced stage cohorts were 0.752 and 0.796, respectively (Figure 6A). According to PAM50 classification, we divided the BRCA patients into four subtypes: luminal A, luminal B, HER2+ and basal-like. Kaplan–Meier and ROC analysis shown that there was too few results in no statistical significance in Kaplan–Meier analysis without the cases of HER2 patients, the survival rate of patients in the low-risk group was greatly improved compared to the high-risk group (*p* < 0.05), and the 6 DNA methylation markers had high predictive performance (AUC > 0.70) (Figure 6B). Chemotherapy is one of the main treatments for BRCA. Considering the influence of drug treatments on the phenotype of patients, we divided breast cancer patients into chemotherapy group and non-chemotherapy group. Our biomarker performed well in both groups, with the patients in the low-risk group shown a better trend of OS (p < 1 × 10^−3^, AUC >0.65) (Figure 6C). In addition, many prognostic markers of BRCA have been reported: CD24, EGFR, CXCR 4 genes can predict the metastasis and prognosis of breast cancer. At the same time, Zhang et al. reported that the DNA methylation of 12 genes could be used as a prognostic marker of BRCA (Zhang et al., 2015). Tao et al. reported that seven differentially DNA methylation sites could be used as a novel prognostic biomarker for BRCA (Tao et al., 2019). In order to determine whether our biomarkers had a better ability to predict patient survival than known biomarkers, ROC analysis of other biomarkers was performed in the same way in the validation cohort. The results shown that the AUC of these 6 DNA methylation markers was higher than that of all other known biomarkers in the validation cohort. The results of ROC analysis showing that the 6 DNA methylation sites are better markers and provide better stability and reliability in predicting the OS of BRCA patients (Figure 6D).

### GSEA Analysis 6 DNA Methylation Site Driven Gene Related Pathway

**Figure 7:**
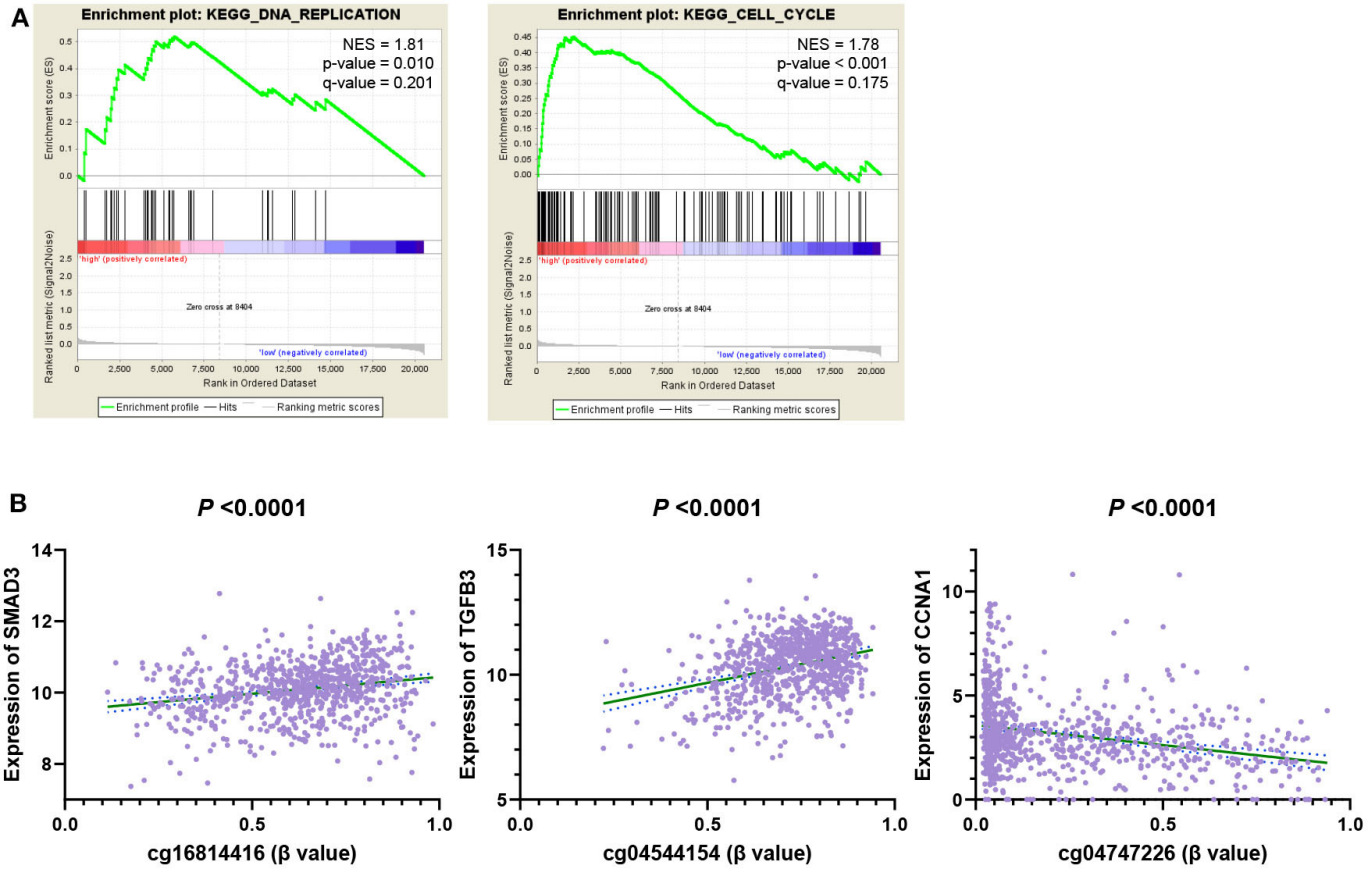
GSEA analysis 6 DNA methylation site driven gene related pathway. **(A)** GSEA KEGG Pathways enrichment analysis genes of high-risk individuals. **(B)** The correlation between 6 CpG markers and genes in the enrichment pathway.

The above analysis shows that our 6 CpG sites could distinguish between the high and low risk groups of breast cancer. In order to explore the mechanism, we conducted the Gene Set Enrichment Analysis (GSEA) KEGG analysis on high and low-risk individuals to explore the possible pathways of 6 CpG sites. We found that the gene expression of the high-risk population identified by the 6 CpG sites model was mainly enriched in the biological behaviors of DNA replication and cell cycle (Figure 7A). We then calculated the correlation between these 6 CpG sites and the genes in the two pathways and found that these 6 CpG sites had significant correlation with many genes of the two pathways (Figure 7B, Supplementary Table 8). This suggested that the underlying biological mechanism of the 6 CpG sites model we found may be related to DNA replication and cell cycle.

## Discussion

Previous studies have suggested that mutations in genes leading to changes in DNA sequences, activation of oncogenes, and inactivation of tumor suppressor genes are major mechanisms of tumorigenesis (Gough et al., 1990; Hahn and Weinberg, 2002; Domchek et al., 2013; Ablain et al., 2018; Poillet-Perez et al., 2018). With the deepening of research on cancer, researchers have found that abnormal regulation mechanisms other than DNA sequences, that is, epigenetic changes also play a key role in tumorigenesis and development (Berdasco and Esteller, 2010). The occurrence and development of breast cancer is a multi-step, multi-stage process, which is considered to be the result of accumulation of genetic and epigenetic variations (Widschwendter et al., 2018). Therefore, it is reasonable and valuable to study the epigenetic mechanisms in the progression of breast cancer to identify clinically applicable biomarkers.

In this study, we systematically analyzed the whole genome methylation data and gene expression data of breast cancer. By comparing BRCA, normal tissue and non-BRCA cancer, we identified seven methylation sites as BRCA specific diagnostic biomarkers. BayesNet model was constructed to predict the diagnosis of BRCA. The sensitivity was 94.2%, and the accuracy was 95.2%. Our 7 CpG sites diagnostic biomarkers had better sensitivity and specificity than most of the previously reported biomarkers and had been verified in a variety of databases. Finally, the abnormal expression of several related genes was verified through experiments. These results provide new insights into the role of DNA methylation in the diagnosis of breast cancer.

Ideal diagnostic biomarkers should be highly sensitive to detect BRCA at an early stage; specific to BRCA and not found in other cancers; measured by non-invasive and cost-effective techniques; and in different populations authenticating. Here, we found 7 BRCA specific differentially methylated sites, which are superior to the widely used serum biomarker CA125 in sensitivity and specificity. However, we have not used non-invasive biological samples to verify their diagnostic ability. In order to solve this problem, we will continue to develop a technique for detecting the methylation level of cell-free ctDNA in serum. Then we will verify the consistency of methylation in tissues and blood and verify the prediction ability of biomarkers by detecting DNA methylation in blood.

In routine clinical practice, some clinicopathological features are used to assess possible prognosis in breast cancer patients, such as tumor size, histological grade, tumor stage, lymph node metastasis, etc. Second, different molecular subtypes of breast cancer also suggest different prognosis. Studies have shown that triple-negative breast cancer tends to have higher tumor grades, higher risk of lymph node metastasis or distant metastasis, and relatively lack of effective treatments, resulting in lower tumor-free survival rate (Liedtke et al., 2008). The results shown that our signature was in dependent of the tumor stage, molecular subtype, or medication.

In addition, Cox regression analysis was carried out on six different methylation sites. Kaplan Meier and ROC analysis shown that each DNA methylation site could also distinguish high-risk and low-risk patients, but the prediction performance was lower than the combination of these 6 DNA methylation sites in the validation cohort, suggesting that a single methylation site may play a role in prognosis prediction, and the combination of methylation sites may provide a better potential to predict OS in BRCA patients.

In summary, our study demonstrated the role of methylation profiles in the diagnosis and prognosis of BRCA. We identified BRCA specific methylation markers to distinguish BRCA tissues from normal tissues. Moreover, our study can also distinguish breast cancer from other cancers. At the same time, we found the BRCA prognostic markers, stratification analysis by clinical stage, tumor types, and chemotherapy retained statistical significance.

## Data Availability Statement

Publicly available datasets were analyzed in this study. This data can be found here: https://cancergenome.nih.gov, https://www.ncbi.nlm.nih.gov/geo/.

## Ethics Statement

The studies involving human participants were reviewed and approved by Ethics Committee of China Medical University belongs to the China Medical University. The patients/participants provided their written informed consent to participate in this study.

## Author Contributions

MZ conceived and designed the study. YiW provided help for the specific ideas of the article. The data were analyzed by YaW and LJ. XL provided part of the code of the R Language. HG, MW, and LZ reviewed and edited the manuscript. All authors read and approved the manuscript.

## Conflict of Interest

The authors declare that the research was conducted in the absence of any commercial or financial relationships that could be construed as a potential conflict of interest.

## Supplementary Material

Supplementary Figure 1 The correlation between 7 DMSs and corresponding genes. Pearson correlation test was used.

Supplementary Figure 2 Unsupervised cluster analysis of 7 DMSs in BRCA and adjacent tissues.

Supplementary Table 1 Profile of healthy blood sample (GSE69270).

Supplementary Table 2 Differentially methylated sites in the blood (GSE69270).

Supplementary Table 3 The profile of GSE66695.

Supplementary Table 4 The profile of GSE60185.

Supplementary Table 5 The profile of GSE78754.

Supplementary Table 6 The DMSs were significantly correlated with corresponding gene expression.

Supplementary Table 7 DNA methylation sites associated with OS significant.

Supplementary Table 8 Genes significantly related to 6 CpG sites on DNA replication and cell cycle pathways.

## Abbreviations

BRCA: breast cancer
GBM: Glioblastoma multiforme
BLCA: Bladder Urothelial Carcinoma
LIHC: Liver hepatocellular Carcinoma
HNSC: Head and Neck squamous cell carcinoma
CESC: Cervical squamous cell carcinoma and endocervical adenocarcinoma
LUAD: Lung Adenocarcinoma
LUSC: Lung Squamous Cell Carcinoma
COAD: Colon adenocarcinoma
READ: Rectum adenocarcinoma
TCGA: Cancer Genome Atlas
GEO: Gene Expression Omnibus
OS: overall survival
DMS: differentially methylated site
CA125: Cancer antigen 125
ctDNA: cell-free tumor DNA
FDR: false discovery rate
KEGG: Kyoto Encyclopedia of Genes and Genomes
GSEA: Gene Set Enrichment Analysis
KNN: k-Nearest Neighbor
RSEM: RNA-Seq by Expectation-Maximization
PPI: Protein-Protein Interaction
HR: hazard ratio
CI: confidence interval
AUC: area under the curve
GO: gene ontology
FC: fold change.
