# Supplementary figures and images for: Integrative Analysis of DNA Methylation and Gene Expression to Determine Specific Diagnostic Biomarkers and Prognostic Biomarkers of Breast Cancer
Source: Front Cell Dev Biol. 2020 Dec 7;8:529386. doi: 10.3389/fcell.2020.529386 (PMC7750432; doi:10.3389/fcell.2020.529386)

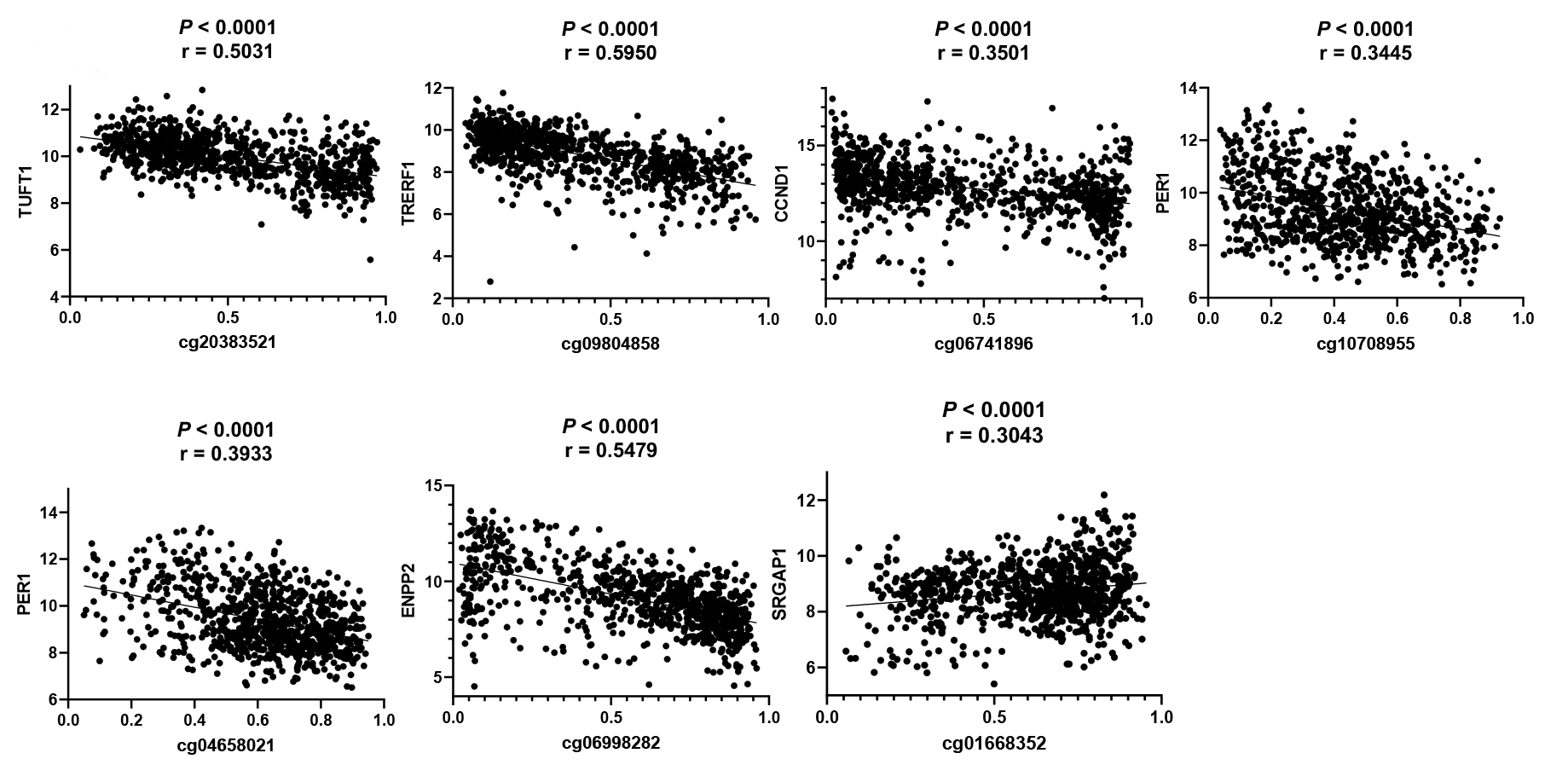

Supplement: Supplementary Figure 1 — The correlation between 7 DMSs and corresponding genes. Pearson correlation test was used. [file Image_1.TIF]

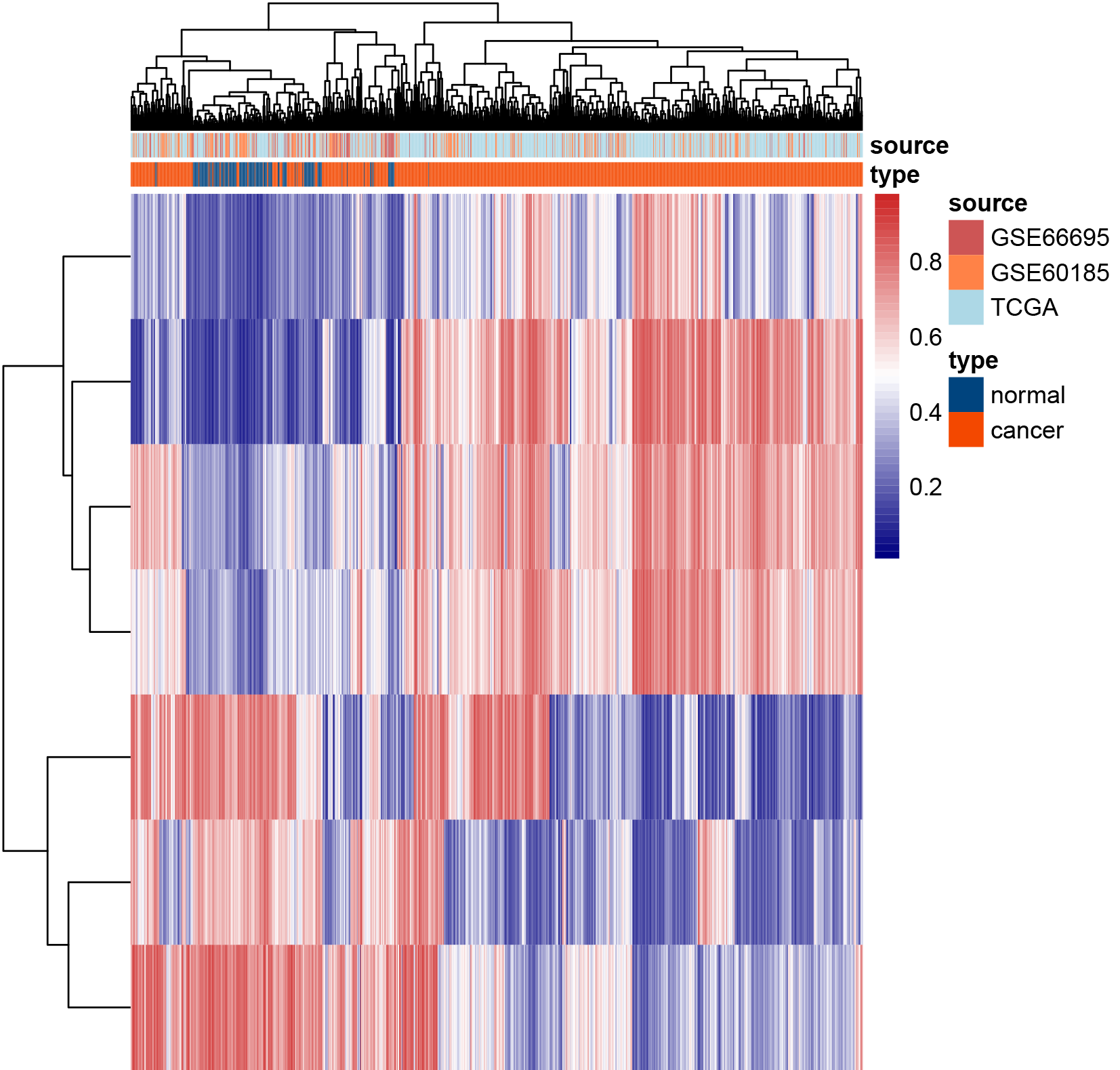

Supplement: Supplementary Figure 2 — Unsupervised cluster analysis of 7 DMSs in BRCA and adjacent tissues. [file Image_2.TIF]
